# Markers of Prognosis for Early Stage Cervical Cancer Patients (Stage IB1, IB2) Undergoing Surgical Treatment

**DOI:** 10.3389/fonc.2021.659313

**Published:** 2021-06-02

**Authors:** Chen Xu, Tie Ma, Hongzan Sun, Xiaohan Li, Song Gao

**Affiliations:** ^1^ Department of Radiology, Shengjing Hospital of China Medical University, Shenyang, China; ^2^ Liaoning Provincial Key Laboratory of Medical Imaging, Shengjing Hospital of China Medical University, Shenyang, China; ^3^ Department of Pathology, Shengjing Hospital of China Medical University, Shenyang, China; ^4^ Department of Obstetrics and Gynecology, Shengjing Hospital of China Medical University, Shenyang, China

**Keywords:** cervical cancer, positron-emission tomography, computed tomography, human papilloma virus, prognosis

## Abstract

**Background:**

For individuals with cervical cancer, large tumor volume, lymph node metastasis, distant metastasis, and parauterine infiltration are usually associated with a poor prognosis. Individuals with stage 1B1 and 1B2 cervical cancer usually do not have these unfavorable prognostic factors. Once the disease progresses, the prognosis becomes extremely poor. Therefore, investigating the prognostic markers of these cervical cancer patients is necessary for treatment.

**Methods:**

This retrospective study included 95 cervical cancer patients treated with surgery. The patients were divided into progressor and non-progressor groups according to postoperative follow-up results. T-test (or Mann−Whitney U test), chi-squared test (or Fisher’s exact test) and receiver operating characteristic (ROC) curves were used to evaluate imaging, hematology, and clinicopathological index differences between the two groups. Cox analysis was performed to select the independent markers of progression-free survival (PFS) when developing the nomogram. Validation of the nomogram was performed with 1000 bootstrapped samples. The performance of the nomogram was validated with ROC curves, generated calibration curves, and Kaplan-Meier and decision curve analysis (DCA).

**Results:**

Cervical stromal invasion depth, lymphovascular space invasion (LVSI), human papilloma virus (HPV-16), Glut1, D-dimer, SUVmax and SUVpeak showed significant differences between the two groups. Multivariate Cox proportional hazard model showed SUVpeak (p = 0.012), and HPV-16 (p = 0.007) were independent risk factors and were used to develop the nomogram for predicting PFS. The ROC curves, Kaplan-Meier method, calibration curves and DCA indicated satisfactory accuracy, agreement, and clinical usefulness, respectively.

**Conclusions:**

SUVpeak level (≥7.63 g/cm3) and HPV-16 negative status before surgery were associated with worse PFS for patients with cervical cancer. Based on this result, we constructed the nomogram and showed satisfactory performance. Clinically, individualized clinical decision-making can be performed on patients based on this result.

## Introduction

Cervical cancer has the second highest incidence of female malignant tumors (1). The International Federation of Gynecology and Obstetrics (FIGO) cancer staging system is used in the formulation of treatment and prognosis plans for patients with cervical cancer. In the recently updated 2018 FIGO staging, it is noteworthy that, assessment of the abdominopelvic retroperitoneal lymph nodes was included in the FIGO system (2–4). Regardless of parametrial infiltration and tumor size, the presence of nodal metastases now indicates stage IIIC. Radical hysterectomy with lymphadenectomy will be effective for those cervical cancer patients (stage 1B1, 1B2) with the following characteristics: invasive carcinoma confined to the uterine cervix, no more than 5 mm invasion, tumor size < 4 cm in its greatest dimension and no lymph node metastasis. Fortunately, these patients often do not need postoperative adjuvant radiotherapy, since, of course, radiotherapy has serious side effects (5). However, although the prognosis of cervical cancer patients with these features is excellent, if they relapse, then their prognosis is very poor (6). Therefore, identifying those cervical cancer patients who are prone to recurrence after surgery is of great significance, since they will require postoperative adjuvant treatment such as radiotherapy and chemotherapy, as well as closer follow-up, to reduce the chance of recurrence and death.

The following three methods were used in predicting the prognosis of cervical cancer: 1) evaluation of pathological characteristics obtained from postoperative pathological specimens, including aberrant molecular signaling pathway proteins; 2) preoperative imaging; and 3) hematological examination.

It is well known that pathological features such as age, tumor stage and grade, cervical stromal invasion depth, lymphovascular space invasion (LVSI); and preoperative high-risk human papillomavirus (HR-HPV) statue were important factors in the prognosis of cervical cancer. Different molecular factors involving loss of tumor suppressor genes and aberrant molecular signaling pathways, such as TP53-induced glycolysis and apoptosis regulator (TIGAR), cytokine involved primarily in angiogenesis (Vegf-A), mammalian facilitative glucose transporter family (Glut-1), epithelial mesenchymal transition related protein (E-cadherin), immune-linked factor by triggering pro-inflammatory immune-associated reactions (Cox-2), the extracellular matrix molecule (Tenascin-C), have recently been identified in the pathogenesis of cervical cancer (7–11). The impact of these proteins on cervical cancer prognosis needs further investigation.

^18^F-FDG PET/CT, a functional imaging technique, provides quantified metabolic information (the mean and maximum standardized uptake values (SUV_mean_ and SUV_max_), metabolic tumor volume (MTV) and total lesion glycolysis (TLG))and has a well-established role in the management of patients with cervical cancer (12–14). Many studies have shown that hematological parameters such as hemoglobin (Hb), coagulation indexes [D-dimer, fibrinogen (Fg)], tumor marker for squamous cell carcinoma [squamous cell carcinoma antigen (SCCA)], and novel systemic inflammation response index (SIRI) are potential prognostic marker of malignant tumors (15–19).

In this article, our aim was to find independent prediction parameters from these parameters and establish and validate a nomogram to predict the progression-free survival (PFS) of patients with cervical cancer.

## Materials and Methods

### Population Characteristics

Patients deemed to have a high suspicion of cervical cancer with strong evidence from PET/CT (PET/CT examinations were performed within one week before treatment) were retrospectively enrolled from January 1^st^, 2013, to December 31^th^, 2015 with the following criteria: (1) The patient underwent surgery (open surgery) and was confirmed as cervical squamous cell carcinoma by postoperative pathological results; (2) According to FIGO2018 pathological staging, the patient was confirmed to be stage IB1 or IB2; (3) Patients who had not undergone other treatments without surgery, and (4) Patients with complete follow-up records.

The Institutional Review Boards at the local institutions endorsed the study. We implemented it in accordance with the ethical standards of the 1964 Helsinki Declaration and subsequent amendments, and gave up informed consent of all participants.

### Surgical Protocol

Numerous studies have compared the effect of the Minimally Invasive and Abdominal Radical Hysterectomy for cervical cancer (20–22). The conclusion shows that for early cervical cancer the minimally invasive surgery (laparoscopic or robot-assisted radical hysterectomy) has a higher recurrence rate than open surgery. In recent years, patients of our hospital have also accepted open abdominal surgery as the main surgical method. Therefore, the patients included all received the open abdominal Type-C radical hysterectomy. The incision was located on the lower abdomen. The resection scope included the uterus, parauterine, upper vagina, and partial tissues around the vagina and pelvic lymph nodes. A sufficient length of adjacent connective tissues, including the front vesicocervix ligament (anterior and posterior lobes), the lateral main ligament, the posterior uterosacral ligament and rectovaginal ligament, also should be removed. The ovary was selectively removed according to the patient’s clinical conditions and willingness. The resection of lymph nodes involved the obturator lymph nodes and the internal, common, and external iliac vessel lymph nodes. Para-aortic nodes should be removed only in the following cases: (1) The preoperative PET/CT showed that there were suspicious metastatic lymph nodes in the para-aortic area [SUVmax of lymph nodes is higher than the background metabolism level (23, 24)]; and (2) the possibility of metastasis in the pelvic or para-aortic lymph nodes was suspected intra-operatively.

### Tumor Recurrence Prediction and Patient Follow-Up

PFS was defined as the time interval between the date of surgery and the date at which the first recurrence of disease was confirmed. Starting from the completion of the surgery, follow-up examinations (chest CT, abdominal CT and pelvic MR) were performed approximately every 3 months for the first 2 years, and then every 6 months or 1 year for the next 3 years. Recurrent disease was defined as local recurrence, metastasis of pelvic or para-aortic lymph nodes, and metastases in distant organs following surgery. For patients with abnormal follow-up examinations results, PET/CT or biopsy was recommended, and the results used as the standard for recurrence. The end of the follow-up time is December 31^th^, 2020.

### Pathologic Diagnosis

A pathologist with more than 10 years of experience evaluated sections using HE staining and immunohistochemistry (IHC) of CD31 and D2-40. The following results based on postoperative pathology report were obtained: tumor differentiation grade, stage, lymph node metastasis, LVSI, cervical stromal invasion depth and histologic tumor type.

Tumor sections were obtained from our hospital pathology department and we purchased antibodies from Abcam (Shanghai, China). In addition, Cox-2, E-cadherin, Tenascin-C, Glut-1, Tigar, and Vegf-A protein expression were studied based on IHC, which was performed using the Leica BOND-MAX system (Leica Biosystems, Shanghai, China). Thorough mixing was done with the following polyclonal antibodies: Cox-2 at 1:100 dilution; E-cad at 1:700 dilution; Tenascin-C at 1:100 dilution; Glut-1 at 1:500 dilution; Tigar at 1:300 dilution; and Vegf-A at 1:50 dilution. Then the tissue section was sealed after the Imaging Mass Cytometry (IMC) was finished, the section was placed under the lens of a scanner (Pannoramic MIDI, 3D Histech), and moved gradually, imaging while moving and then scanning and imaging all the tissue information on the tissue section to form a file. After image scanning was completed, the DensitoQuant software application in the QuantCenter (QuantCenter is a piece of analysis software for Pannoramic viewer) automatically recognized and set all dark browns on the tissue section as strongly positive, brown-yellow as moderately positive, light yellow as weakly positive, and blue cell nuclei as negative. Furthermore, for each tissue, strongly positive, moderately positive, weakly positive and negative areas, and the percentage of positive areas, were identified. A negative expression was defined by a < 10% positive expression.

### HPV Examination

For HPV detection we used an AB I2Prism7000 PCR detector, a Hybrid-Max medical nucleic acid molecular hybridizer (AB, USA), and a PCR kit (Qiagen, USA). HPV detection used an AB I2Prism7000 PCR detector, a Hybrid-Max medical nucleic acid molecular hybridizer (AB, USA), and a PCR kit (Qiagen, USA). Fifteen types of the HR-HPV types (HPV16, 18, 31, 33, 34, 39, 45, 51, 52, 53, 56, 58, 59, 66, and 68) were detected and diagnosed by a pathologist with more than 10 years of diagnostic experience.

### Hematology Diagnostic Criteria

Included in the novel systemic inflammation index were the following: NLR, defined as neutrophil-to-lymphocyte ratio; LMR, defined as mononuclear cell-to-lymphocyte ratio; and SII, defined as ratio of product of platelets and neutrophils to lymphocytes (25, 26). Evaluation was undertaken of the relationship between the novel systemic inflammation index and prognosis based on the ROC curve and calculation of the optimal cut-off value for meaningful indexes. Anemia was defined as a hemoglobin count below 11.0 g/dL. The optimal D-dimer, Fg and SCCA cut-off range for assessing prognosis was 0-0.5 mg/L, 2-4 g/L, and 0-2.5 ng/ml. These cut-off ranges were selected as they have been recognized as standard pathological definitions.

### PET/CT Scanning and Image Acquisition

All patients were fasted for more than 6 hours before the 18F-FDG PET/CT examination, blood glucose levels were controlled below 7 mmol/L, and 18F-FDG was injected with the patient in a quiet state from 3.70 to 5.55 MBq/kg. After 45–60 minutes, 18F-FDG PET/CT was performed. The patient was placed in a GE Discovery Elite scanner (GE Healthcare, USA) with the scan ranging from the skull top to the middle of the thigh (120s/bed). The thickness of the CT scan layer was 3.75 mm, the tube voltage was 120–140 keV, and the tube current was 80mA. PET images were reconstructed using an Iterative adaptive algorithm.

### Image Analysis

Transmitting all ^18^F-FDG PET/CT images to GE AW4.6 workstation (GE Healthcare, USA), 2 radiologists with more than 5 years of radiodiagnostic experience used PET volume computer-assisted reading (PET VCAR) software to perform imaging analysis. After outline of a lesion region of interest (ROI) at the cross-sectional level of the largest area of the tumor, the fused-PET software automatically calculated SUV_mean,_ SUV_max_, MTV and TLG of the entire tumor. MTV was calculated by a 40% SUV_max_ threshold (27, 28).

### Statistical Analysis

The patients were divided into a positive and a negative group according to prognosis. Differences in patient characteristics between the groups were compared with the t-test (Mann-Whitney U test, if not normal distribution) or chi-squared (Fisher’s exact test, if not the number of assumptions necessary). For quantitative variables, receiver operating characteristic (ROC) curves were generated to assess the area under the ROC curve (AUC) for differentiating prognosis. We used the cut-off threshold values for differentiating these meaningful quantitative variables. The Kaplan-Meier method was used to build survival functions and the log-rank test was used to compare survivals for all variables. Prognostic variables were evaluated by univariate and multivariate Cox proportional hazard model (variables with p<0.1 in the univariate Cox proportional hazard model were used in the multivariate analysis). In multiple testing, a correction was performed, based on the Benjamini-Hochberg procedure. Finally, these final prognostic variables were incorporated to construct the nomogram. The nomogram adopted the 1-, 2-, and 5-year PFS as primary endpoints. Validation of the nomogram was performed with 1000 bootstrapped samples, that is, we adopted the method of bootstrapped samples to set up a control group, randomly selected 1000 times from the existing samples, arbitrarily once, and then a final time to get the original sample number (95). The predictive capability was evaluated by ROC analysis. Generated calibration curves were used to visualize the difference between the predicted and actual 1-, 2-, and 5-year PFS. Decision curve analysis (DCA) was introduced to evaluate clinical utility of the nomogram (29). Based on the median of the total scores, a risk stratification system was developed, and the cervical cancer patients were divided into two risk subgroups, including high-risk and low-risk groups, and the Kaplan-Meier method and log rank test were used to compare the differences between the two subgroups. The statistical analysis was conducted by MedCalc (version 15.2.2), SPSS (version 22.0), R (version 3.5.3), and p<0.05 indicated a significant statistical difference (if not specified).

## Results

### Survival and Disease Control

**Figure 1:**
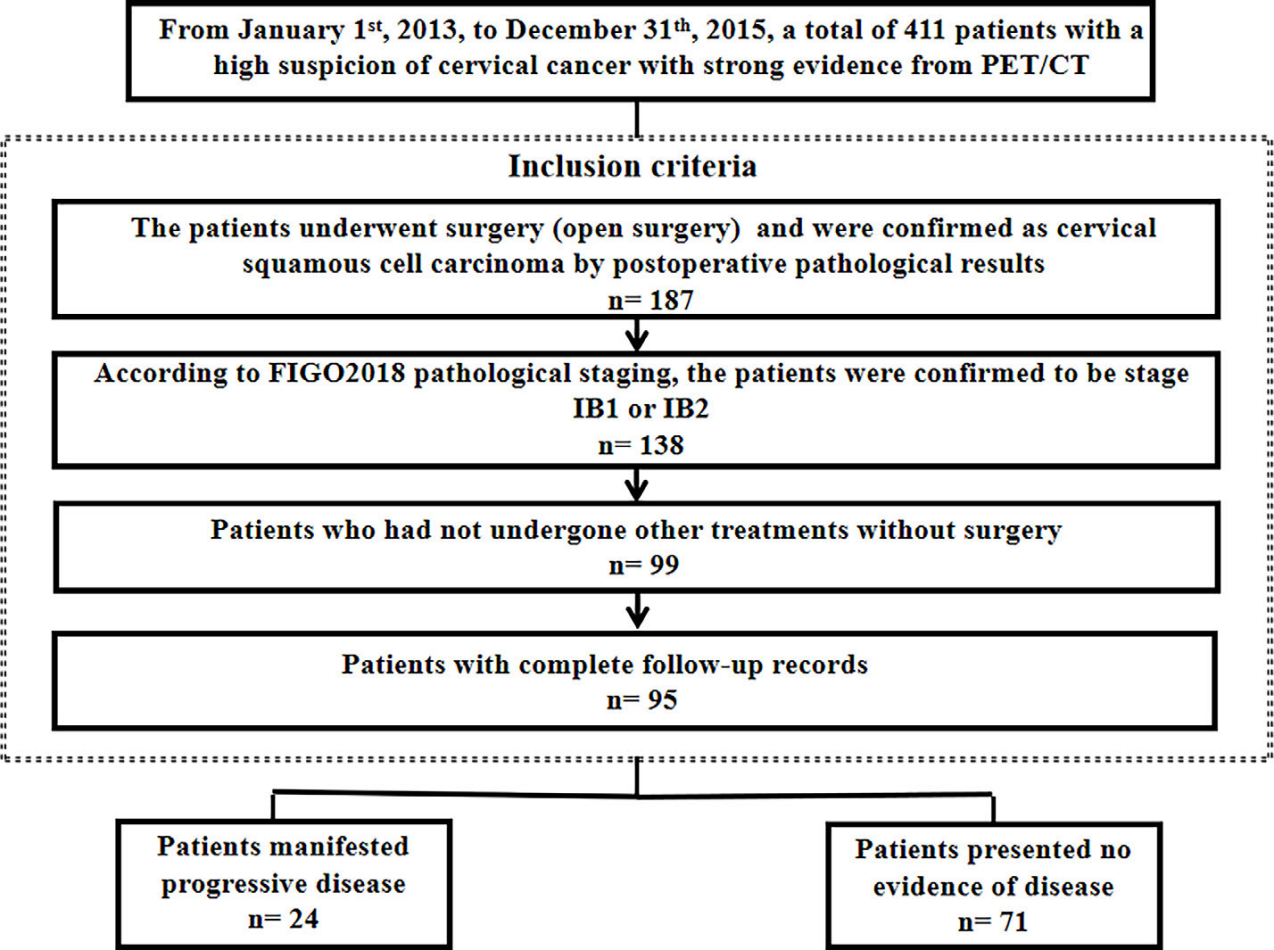
Patient selection.

The median follow-up was 62.8 months (range, 2-96). A total of 95 patients were enrolled: 24 (25.3%) patients manifested progressive disease and 71 (74.7%) patients presented no evidence of disease (**Figure 1**).

### The Relationship Between Disease Progression and Clinicopathological Characteristics in Patients

The clinicopathological characteristics of the patients are summarized in **Table 1A**. Cervical stromal invasion depth (chi-squared test, p=0.046; log-rank test, p=0.032), LVSI (chi-squared test, p=0.032; log-rank test, p=0.008) and HPV-16 (chi-squared test, p=0.001; log-rank test, p<0.001) showed differences between patients with and without disease progression. The remaining indicators had no significant correlation.

**Table 1a:** Patients’ clinicopathological characteristics.

Feature	Total	Nun-progressor	Progressor	Value - p		
				chi-squared	log-rank	t-test/U-test
No. of patients	95	71	24			
Mean age (years)	49.40 ± 9.26	49.06 ± 9.14	50.42 ± 9.73	/	/	0.968
FIGO stage (2018):						
Ib1	11 (11.6%)	9	2	0.565	0.531	/
Ib2	84 (88.4%)	62	22			
Differentiation grade:						
Well-moderately differentiated	86 (90.5%)	64	22	0.825	0.748	/
Poorly differentiated	9 (9.5%)	7	2			
Cervical stromal invasion depth:						
< ½	36 (37.9%)	31	5	0.046	0.032	/
≥ ½	59 (62.1%)	40	19			
LVSI						
Negative	71 (74.7%)	57	14	0.032	0.008	/
Positive	24 (25.3%)	14	10			
HR-HPV:						
Negative	19 (20%)	7	12	0.194	0.176	/
Positive	76 (80%)	17	59			
HPV-16						
Negative	43 (45.3%)	25	18	0.001	<0.001	/
Positive	52 (54.7%)	46	6			
HPV-18						
Negative	88(45.3%)	67	21	0.266	0.226	/
Positive	7(45.3%)	4	3			

FIGO stage (2018): Postoperative pathological staging; LVSI, lymphovascular space invasion; HR-HPV, high-risk human papillomavirus; Normal distribution data, means ± standard deviations; t-test. Non-normal distribution data, medians and interquartile ranges; U-test. /, No statistics.

### The Relationship Between Disease Progression and Protein Expression in Cancer Tissue

Chi-squared test and Kaplan-Meier method showed that patients with Glut1-negative had a statistically significant better clinical outcome than patients with Glut-1-positive (chi-squared test, p=0.039; log-rank test, p=0.039) (**Table 1B**). The remaining protein indicators were not statistically significant.

**Table 1b:** Patients’ protein expression.

Feature	Total	Nun-progressor	Progressor	Value - p		
				chi-squared	log-rank	t-test/U-test
No. of patients	95	71	24			
Vegf-A:						
Negative	49 (51.6%)	37	12	0.858	0.837	/
Positive	46 (48.4%)	34	12			
Glut1:						
Negative	41 (43.2%)	35	6	0.039	0.039	/
Positive	54 (56.8%)	36	18			
E-cadherin:						
Negative	52 (54.7%)	40	12	0.590	0.487	/
Positive	43 (45.3%)	31	12			
Cox-2						
Negative	66 (69.5%)	48	18	0.496	0.547	/
Positive	29 (30.5%)	23	6			
Tenascin-C:						
Negative	50 (52.6%)	35	15	0.264	0.332	/
Positive	45 (47.4%)	36	9			
Tigar:						
Negative	30 (31.6%)	22	8	0.831	0.848	/
Positive	65 (68.4%)	49	16			

### The Relationship Between Disease Progression and Hematology-Related Parameters in Patients

Chi-squared test and Kaplan-Meier method showed only D-dimer was related to disease progression and PFS (**Table 1C**). The ROC curve showed that no novel systemic inflammation index was statistically significant for disease progression.

**Table 1c:** Patients’ hematology-related parameters.

Feature	Total	Nun-progressor	Progressor	Value - p		
				chi-squared	log-rank	t-test/U-test
No. of patients	95	71	24			
Hb:						
≥ 110 g/dL	51 (53.7%)	41	10	0.172	0.180	/
<110 g/dL	44 (46.3%)	30	14			
D-Dimer:						
Negative	45 (47.4%)	38	7	0.039	0.033	/
Positive	50 (52.6%)	33	17			
Fg:						
Negative	57 (60.0%)	42	15	0.772	0.691	/
Positive	38 (40.0%)	29	9			
SCCA:						
Negative	58 (61.1%)	45	13	0.424	0.416	/
Positive	37 (38.9%)	26	11			
NLR:	1.92 (1.32, 3.00)	2.09 (1.38, 3.14)	1.54 (1.25, 2.46)	/	/	0.084
LMR:	0.20 (0.16, 0.27)	0.20 (0.17, 0.29)	0.18 (0.14, 0.27)	/	/	0.271
SII:	411.06 (298.67, 710.35)	416.20 (312.26, 710.35)	375.32 (293.99, 579.40)	/	/	0.333

Hb, Hemoglobin; Fg, fibrinogen, SCCA, squamous cell carcinoma antigen; NLR, neutrophil-to-lymphocyte ratio; LMR, mononuclear cell-to-lymphocyte ratio; SII, systemic immunity-inflammation index. Normal distribution data: means ± standard deviations; t-test. Non-normal distribution data: medians and interquartile ranges; U-test. /, No statistics.

### The Relationship Between Disease Progression and PET/CT Parameters in Patients

**Figure 2:**
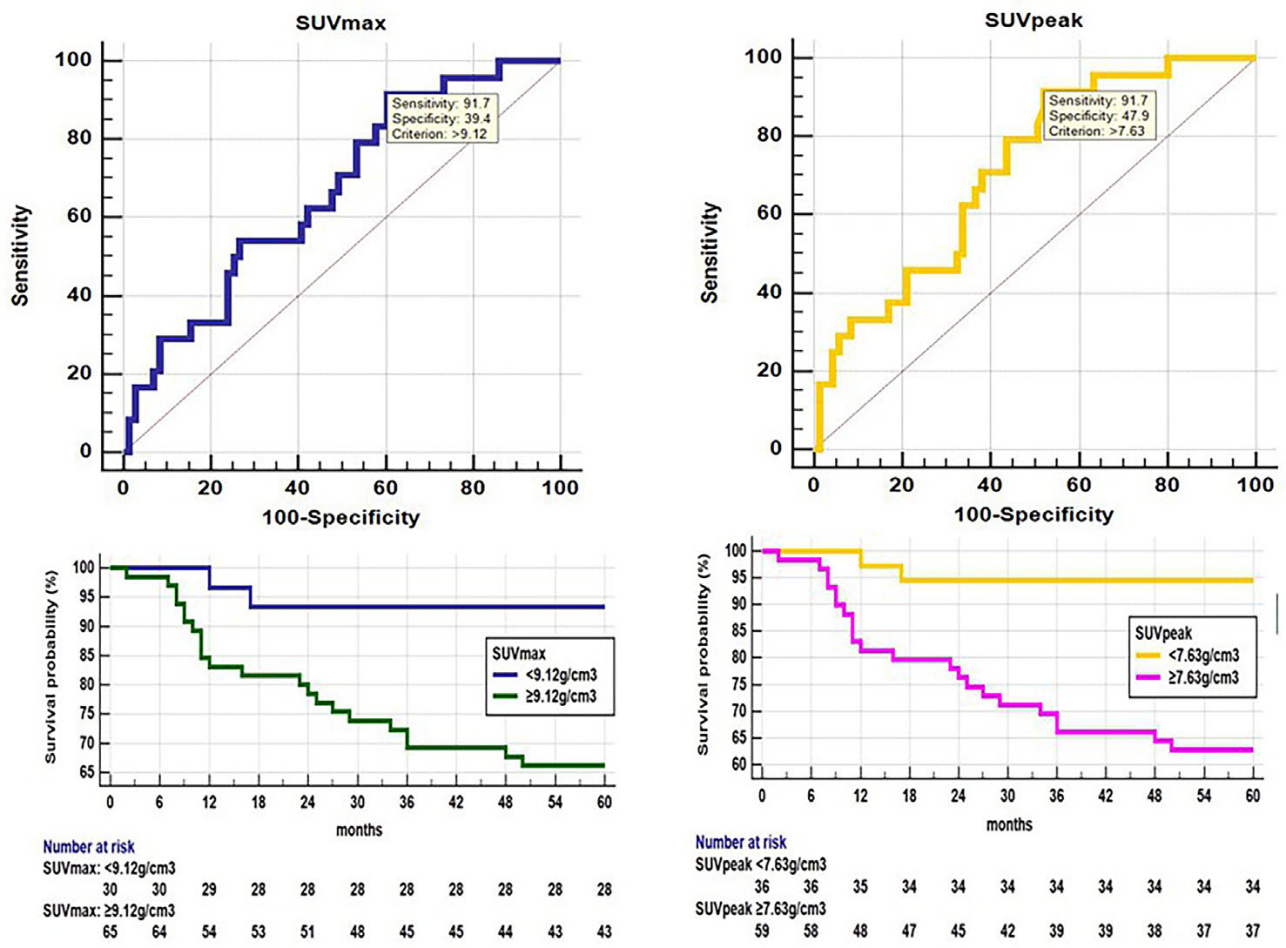
ROC analysis shows that SUV_max_(AUC 0.668, 95% confidence interval (CI) 0.564-0.761, p=0.006) and SUV_peak_ (AUC 0.716, 95% CI 0.615-0.804, p<0.001) had a positive effect on disease progression. The optimal cut-off threshold values for SUV_max_ and SUV_peak_ were 9.12 g/cm^3^ (sensitivity 91.67, specificity 39.44), 7.63 g/cm^3^ (sensitivity 91.67, specificity 47.89), respectively. Kaplan-Meier survival graph shows significantly different PFS between the groups categorized by SUVmax and SUVpeak above and below cut-off value (p<0.05, log-rank test).

SUV_max_ (U test, p=0.014) and SUV_peak_ (U test, p=0.002) showed significant between-group differences (**Table 2**). ROC analysis showed that SUV_max_ (AUC 0.668, p=0.006), SUV_peak_ (AUC 0.716, p<0.001) had a positive effect on predicting disease progression (**Figure 2**). The optimal cut-off threshold values for SUV_max_, SUV_peak_ were 9.12 g/cm^3^ (sensitivity 91.67, specificity 39.44) and 7.63 g/cm^3^ (sensitivity 91.67, specificity 47.89), respectively. Kaplan-Meier method showed the DFS rates of patients exhibiting high SUV_max_ and SUV_peak_ of the primary tumor were significantly lower than those of patients exhibiting low SUV_max_ and SUV_peak_ of the primary tumor (p=0.006 and p=0.001, respectively) (**Figure 2**).

**Table 2:** Patients’ PET/CT parameters.

	Nun-progressor	Progressor	Value - p
No. of patients	71	24	
SUV_max_ (g/cm^3^)	10.87 (7.58, 13.66)	13.29 (10.57, 20.80)	0.014
SUV_mean_ (g/cm^3^)	6.23 (4.17, 8.31)	7.78 (5.57, 10.15)	0.149
SUV_peak_ (g/cm^3^)	8.27 (5.29, 11.47)	10.12 (8.84, 18.69)	0.002
MTV (cm^3^)	10.20 ± 6.28	10.31 ± 6.50	0.876
TLG (g)	47.70 (32.72, 87.22)	54.10(38.30, 153.19)	0.014

Normal distribution data: means ± standard deviations; t-test.

Non-normal distribution data: medians and interquartile ranges; U-test.

### Univariate and Multivariate Cox Proportional Hazard Model

Univariate Cox proportional hazard model showed that cervical stromal invasion depth (p=0.041), LVSI (p=0.018), HPV-16 (p=0.001), Glut1 (p=0.048), D-dimer (p=0.040), SUV_max_ (p=0.016) and SUV_peak_ (p=0.005) were associated with PFS. Multivariate Cox proportional hazard model showed that SUV_peak_ (p=0.012), and HPV-16 (p=0.007) were independent risk factors for PFS (**Table 3**).

**Table 3:** Prognostic factors for DFS selected by Cox analysis.

Univariate Cox proportional hazard model: enter variable <0.1Method: Forward: LR						
Variable	Reference	Characteristic		P	HR	95% CI
Cervical stromal invasion depth	<½	≥½		0.041	2.801	1.045-7.507
LVSI	negative	positive		0.018	2.661	1.181-5.998
HPV-16	positive	negative		0.001	4.651	1.844-11.736
Glut1	negative	positive		0.048	2.544	1.010-6.411
D-dimer	negative	positive		0.040	2.511	1.041- 6.056
SUV_max_	<9.12 g/cm^3^	≥9.12 g/cm^3^		0.016	5.901	1.387-25.103
SUV_peak_	<7.63 g/cm^3^	≥7.63 g/cm^3^		0.005	8.022	1.885-34.135
**Multivariate Cox proportional hazard model:Benjamini-Hochberg procedure was applied to the final analyses; enter variable <0.05 Method: Forward: LR**						
Variable	**Reference**	**Characteristic**		**P**	**HR**	**95% CI**
SUV_peak_	<7.63 g/cm^3^	≥7.63 g/cm^3^		0.012	8.342	1.956-35.574
HPV-16	positive	negative		0.007	4.834	1.909-12.239

LVSI, lymphovascular space invasion; HPV, human papillomavirus.

### Nomogram Development and Validation

**Figure 3:**
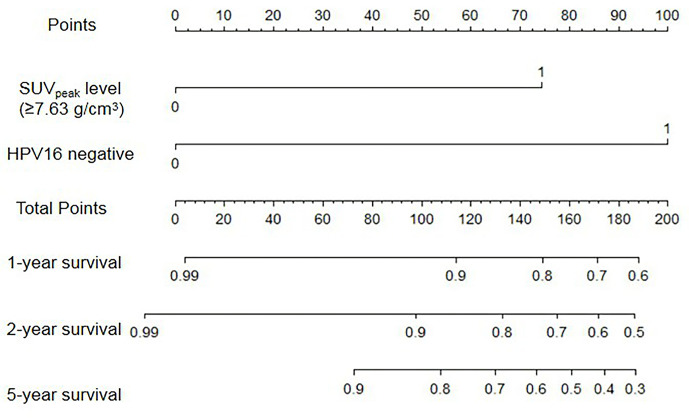
Nomogram for predicting 1-, 2-, and 5-year PFS of cervical cancer.

**Figure 4:**
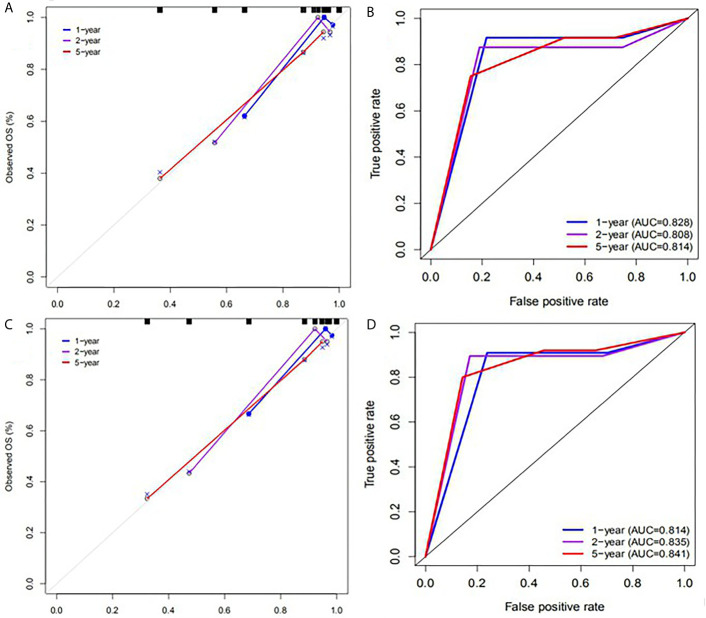
Calibration plots and ROC curves for predicting PFS at 1-, 2-, and 5- year points. **(A)** The calibration plots for predicting PFS in the original model. **(B)** ROC curves of the nomogram for predicting PFS in the original model. **(C)** The calibration plots for predicting PFS in the validation model. **(D)** ROC curves of the nomogram for predicting PFS in the validation model.

**Figure 5:**
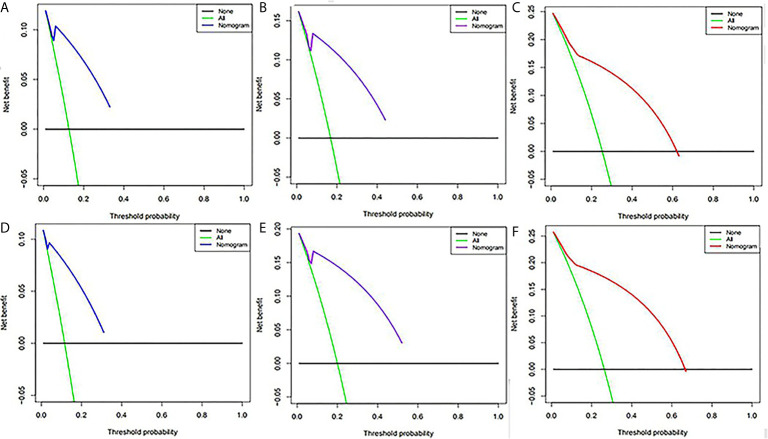
Decision curve analysis of the nomogram for predicting PFS at 1-,**(A)** 2-,**(B)** and 5-year **(C)** points in the original model and PFS at 1-**(D)**, 2-**(E)** and 5-year **(F)** points in the validation model. The percentage of threshold probability was represented by the x-axis, whereas the net benefit was represented by the y-axis, calculated by adding the true positives and subtracting the false positives.

**Figure 6:**
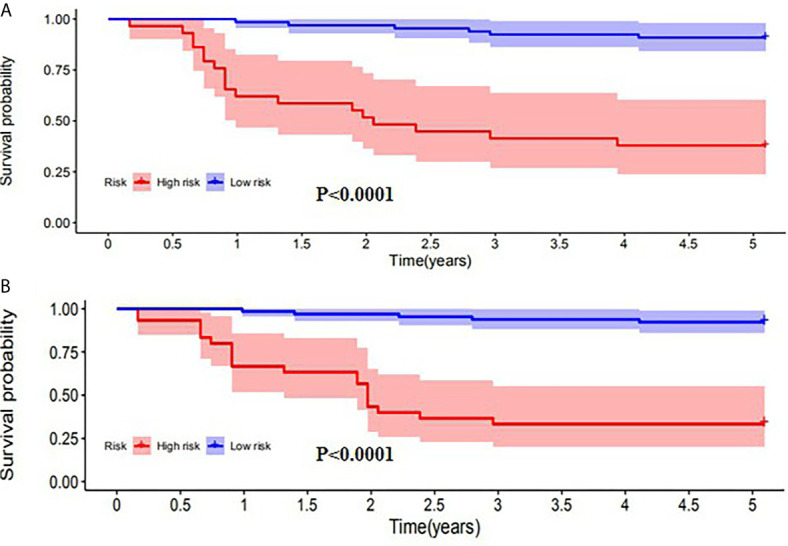
Kaplan-Meier analysis of PFS for patients stratified by the risk stratification system in the original model **(A)** and validation model **(B)**.

SUV_peak_ and HPV-16 were incorporated to develop the nomogram for predicting 1-, 2-, and 5-year PFS. The nomogram showed that SUV_peak_ made the largest contribution to the prognosis, followed by HPV-16 which showed a certain amount of impact on the PFS (**Figure 3**). ROC analysis showed the AUCs at 1-, 2-, and 5-year PFS reached 0.828, 0.808 and 0.814 in the original model, and 0.814, 0.835 and 0.841 in the validation model, respectively (**Figures 4B, D**). Whether in the original model or in the validation model, the calibration curves demonstrated considerable agreement between the nomogram and predicted survival (**Figures 4A, C**). Clinical utility of the nomogram was evaluated by DCA. The nomogram showed enormous positive net benefits across wide ranges of mortality risk in both models, demonstrating its predominant clinical utility in predicting PFS (**Figure 5**). In particular, it has the greatest clinical utility for 5-year PFS in both models. In addition, we calculated the total scores for the original model to build a risk stratification system based on our nomogram, and then distinguished the patients according to the median quantile of total scores into high-risk subgroups and low-risk subgroups. The PFS in the two subgroups was exactly separated by this system (**Figures 6A, B**).

## Discussion

A poor prognosis for patients with cervical cancer is usually associated with a large tumor volume, lymph node metastasis, distant metastasis, and parauterine infiltration. In this study, we explored the prognostic indicators of patients with stage 1B1 and 1B2 cervical cancer without these factors, and established a prognostic nomogram and risk stratification system. Our study showed SUV_max_, SUV_peak_, Glut1, HPV-16, cervical stromal invasion depth, LVSI and D-dimer were associated with PFS. SUV_peak_ and HPV-16 were identified as factors independently impacting disease progression.

The basis of PET imaging is the “Warburg effect”, that is, tumor cells mainly produce ATP through a higher rate of glycolysis compared with normal cells. SUV_max_, a type of PET parameter, reflects the metabolism of the most active part of the lesion. SUV_peak_, another type of SUV, refers to the average SUV within a spherical VOI positioned around the most active metabolism point (30). The glucose uptake of cells is mediated by Glut, and Glut-1 is an important isomer of glucose transporter. Univariate cox analysis shows that SUV_max_, SUV_peak_ and Glut1 have a certain correlation with PFS, but multivariate analysis shows that only SUV_peak_ is considered to be an independent factor, among these three parameters. SUV_max_ is the most commonly used PET parameter in the clinic, but it is a single element measurement value and is susceptible to image resolution and noise. Compared with SUV_max_, the value of SUV_peak_ is more stable and accurate because it is not easy affected by tracking bed position, scanning time and the size of the lesion (31, 32). A study on the survival analysis of patients with cervical cancer showed that among the five classic PET parameters: SUV_max_, SUV_mean_, SUV_peak_, MTV, TLG and six texture features, SUV_peak_ is the most accurate parameter in predicting the disease progression (33). Zhang Le et al. showed that SUV_peak_ had the highest correlation with the clinicopathological features of cervical cancer (34). These all support our results. Compared with other glucose metabolism parameters, SUV_peak_ is more closely related to the prognosis of patients undergoing surgery. It is worth noting that MTV, TLG and PFS are not related in our study. Yoo et al. conducted a prognostic study of cervical cancer at stage I–IV and showed that MTV and TLG are important PET parameters for predicting disease progression (35). The study of Maura et al. and Sangwon et al. had similar findings (36, 37). This may be because the largest diameter of the cancerous lesions in our study subjects was less than 4cm. However, MTV and TLG reflect the overall metabolic burden of the tumor. Therefore, the parameters MTV and TLG related to the tumor volume did not play a major role in our research.

HPV is a genus of papilloma vacuole virus A, belonging to the papovaviridae family. It is a spherical DNA virus that can cause the proliferation of squamous epithelium of human skin and mucosa. High-risk HPV can be detected in most cervical cancer specimens. HPV16 is a common high-risk type of HPV that is most prone to persistent infection and can be detected in about 40% to 60% of patients with cervical cancer (38). However, a study showed that HPV-negative status is associated with a poor prognosis in patients with cervical cancer, which may be related to WNT/β-catenin signaling and non-synonymous somatic mutations (39). Another Meta-Analysis showed the presence of HPV-16 positivity appears to have no significant association with prognosis cervical cancer in PFS. But, eliminating a study with a strong impact on the outcome from the analysis would lead to a conclusion of a worse prognosis of HPV-16 negative in cervical cancer (40). In addition, one study showed that cervical cancer patients infected with HPV16 had a better prognosis than those with any other HPV type (41). These results are roughly consistent with our study, in which HPV16 negativity was associated with worse PFS for those patients with cervical cancer.

Cervical stromal invasion depth is an essential index in the standard pathological report, and represents the invasion status of tumor cells. Lymphovascular space invasion (LVSI) is a common clinical pathological phenomenon of malignant tumors. For tumors to form distant metastases and spread, tumor cells must first enter the circulatory system and spread through blood or lymphatic vessels. Therefore, LVSI is associated with distant metastasis, from a histological perspective (42). D-dimer is a degradation product produced following fibrinolysis, which participates in the coagulation process. Its plasma level can be used as an evaluation index for blood hypercoagulability and as a measure of whether secondary fibrinolytic hyperfunction occurs (43). Our research showed that the above three indicators were related to prognosis, but they were not independent prognostic factors.

Our combined nomogram developed could effectively identify the patients with progressive disease. It is observed that SUV_peak_ is of the most value to prognosis, followed by HPV-16. We established a control model using 1000 bootstrapped samples at the same time. The nomogram of the original group and the control group both showed excellent performance, as was indicated by the ROC curves, generate calibration curves, DCA and Kaplan-Meier method. The ROC curves and Kaplan-Meier method showed that the model had good prediction accuracy, and the calibration curves demonstrated consistency. However, even if the model has high accuracy, patients may not necessarily benefit clinically, and there may be false positives and false negatives. Sometimes it is more beneficial to avoid false positives, and at other times more desirable to avoid false negatives. Since neither situation can be avoided, a method with the greatest net benefit is needed. This is what DCA does. The DCA of our model showed great clinical performance, especially for patients with 5 year-PFS. The capability of the combined nomogram for prediction of progressive disease may facilitate personalized treatment decisions.

This study has a number of limitations. On one hand, this is a retrospective study based on the latest FIGO2018 staging. Because the early follow-up was not optimal, the sample size of our study was not sufficiently large, especially for positive cases, thus further future confirmation of the results is needed. Furthermore, there is a lack of data about several important factors, including some functional sequences of magnetic resonance, proteomics and genomics data, and pathway proteins related to prognosis, such as Hive, Caix, etc. Finally, in our original data, compared with patients with squamous cell carcinoma, the proportion of patients with adenocarcinoma is particularly small. In order to prevent extreme value bias, we only selected patients with squamous cell carcinoma as the research object. In the future, additional types of pathology will be included in our research.

## Conclusion

In conclusion, SUV_peak_ level (≥7.63 g/cm^3^) and HPV16 negative were identified as independent factors and could be associated with poor prognosis for patients with cervical cancer (Stage IB1, IB2). Based on this result, we established a nomogram and risk stratification system, and achieved satisfactory performance and clinical utility. These findings could contribute to test-individualized neoadjuvant treatment. It is worth emphasizing that the stage of cervical cancer of patients selected was only Stage IB1 and IB2. That is, patients with cervical cancer of other stages were not within the scope of this study. At the same time, cervical stromal invasion depth, LVSI, and D-dimer were related to prognosis, but they were not associated with poor DFS in the multivariate analysis. Cautions would be needed when clinical decision-making was made for patients with these risk factors.

## Data Availability Statement

The original contributions presented in the study are included in the article/supplementary material. Further inquiries can be directed to the corresponding authors.

## Author Contributions

CX and SG designed the research. CX, TM, XL and SG performed the research and analyzed results. CX wrote the paper. CX, XL, HS,TM and SG edited the manuscript and provided critical comments. All authors contributed to the article and approved the submitted version.

## Funding

This study was funded by LIAONING Science & Technology Project (2017225012), LIAONING Science Natural Science Foundation (2019-MS-373) and 345 Talent Project.

## Conflict of Interest

The authors declare that the research was conducted in the absence of any commercial or financial relationships that could be construed as a potential conflict of interest.
